# DOA and Polarization Estimation Using an Electromagnetic Vector Sensor Uniform Circular Array Based on the ESPRIT Algorithm

**DOI:** 10.3390/s16122109

**Published:** 2016-12-13

**Authors:** Na Wu, Zhiyu Qu, Weijian Si, Shuhong Jiao

**Affiliations:** Department of Information and Communication Engineering, Harbin Engineering University, Harbin 150001, China; wunahmc@163.com (N.W.); swj0418@263.net (W.S.); jiaoshuhong@hrbeu.edu.cn (S.J.)

**Keywords:** electromagnetic vector sensor array (EVSA), DOA and polarization estimation, ESPRIT algorithm, fourth-order cumulant, uniform circular array, rotation invariant

## Abstract

In array signal processing systems, the direction of arrival (DOA) and polarization of signals based on uniform linear or rectangular sensor arrays are generally obtained by rotational invariance techniques (ESPRIT). However, since the ESPRIT algorithm relies on the rotational invariant structure of the received data, it cannot be applied to electromagnetic vector sensor arrays (EVSAs) featuring uniform circular patterns. To overcome this limitation, a fourth-order cumulant-based ESPRIT algorithm is proposed in this paper, for joint estimation of DOA and polarization based on a uniform circular EVSA. The proposed algorithm utilizes the fourth-order cumulant to obtain a virtual extended array of a uniform circular EVSA, from which the pairs of rotation invariant sub-arrays are obtained. The ESPRIT algorithm and parameter pair matching are then utilized to estimate the DOA and polarization of the incident signals. The closed-form parameter estimation algorithm can effectively reduce the computational complexity of the joint estimation, which has been demonstrated by numerical simulations.

## 1. Introduction

In the past few decades, the direction of arrival (DOA) estimation of incident signals has been demonstrated to play a significant role in array signal processing [1,2,3,4]. Electromagnetic vector sensor arrays (EVSAs) can receive the incident electromagnetic waves in the form of vectors, which include both the polarization domain and the spatial domain information [5,6]. EVSAs have some inherent advantages [7,8,9,10,11] over traditional arrays. Firstly, EVSAs have superior system performances, which the signals can be distinguished based on their polarization characteristics. Secondly, more accurate models of direction finding systems can be established within EVSAs. Finally, EVSAs have stronger ability of anti-fuzzy than scalar sensor arrays, etc.

As a potential solution, EVSA-based techniques have been widely used in many fields, such as radar [12,13], communication [14], sonar [15,16], etc. Various DOA and polarization joint estimation algorithms have been developed recently, including MUSIC [17,18], ESPRIT [19,20,21,22,23], pencil-MUSIC [24,25], Root-MUSIC [26], etc. An enhanced MUSIC algorithm, proposed by Hua, et al. [24], can effectively improve the estimation accuracy. It is quite applicable to arrays with arbitrary geometries. This algorithm, however, involves the spectrum function construction in four dimensions, leading to fairly high computational complexity. ESPRIT algorithm was initially applied to a uniform linear vector sensor array composed of crossed dipoles for multiple-signal joint DOA and polarization parameters estimation in [20,21,22]. Since the ESPRIT-based algorithm is a closed-form estimation method based on eigen-structure, the computational cost is lower than that of MUSIC-based algorithms. However, ESPRIT-based algorithms require the data to possess certain “invariant” structures, inapplicable to the uniform circular EVSAs.

In recent years, characteristics of vector sensors within hypercomplex frameworks, such as quaternions [27], biquaternions [28], and quad-quaternions [29], etc., have been studied extensively. These frameworks retain the vector characteristic of vector sensor arrays, with advantages of a more compact form and better estimation performance for signal subspace. However, quaternions, biquaternions or quad-quaternions can only deal with four-dimensional (4D), eight-dimensional (8D) and sixteen-dimensional (16D) algebras, respectively. Tensors [10,30], however, can be used to deal with arbitrary dimensional algebras meanwhile keep corresponding vector information. It is worth noting that the advanced algebraic theories, such as multivariate algebra and tensor, are typically utilized to establish new models for the output of the array. In general, two types of representative algorithms are still MUSIC-based algorithms (such as Q-MUSIC [31], BQ-MUSIC [32,33], QQ-MUSIC [34], tensor-based MUSIC [35] etc.) and ESPRIT-based algorithms (such as quaternion-ESPRIT [36], tensor-ESPRIT [37] etc.). Therefore, the advantages and disadvantages of the traditional MUSIC and ESPRIT algorithm exist inherently in these MUSIC-based algorithms and ESPRIT-based algorithms.

The selection of array geometry is significant to estimate the DOA and polarization information [38], which determines the estimation accuracy, the computation complexity and the implementation possibility. In general, the EVSAs have the forms of uniform linear patterns [39], uniform rectangular patterns [40,41], L-shaped patterns [42] and uniform circular patterns [43], etc., all of which can be classified into concurrent EVSAs [31,33,34,36] and spatially separated EVSAs (SS-EVSAs) [44,45]. Various constraints such as the carrier profile, the electromagnetic and aerodynamic compatibility, however, should be taken into account comprehensively in practical systems. Uniform circular EVSAs are easier to keep conformal with carriers, and possess the characteristics of smaller radar cross section (RCS) and scan-angle-independent beam width, etc. However, rotation invariant structures cannot be constructed from the array steering matrices, which causes that the ESPRIT algorithm is unfeasible here. In order to estimate the DOA and polarization information by ESPRIT algorithm based on the uniform circular array, a fourth-order cumulant-based ESPRIT algorithm is proposed in this paper.

Since the fourth-order cumulant results in a larger array aperture and is blind to Gaussian processes, conventional array processing techniques generally utilize it to increase the number of localizable sources and improve the estimation accuracy [46]. Here, the proposed algorithm utilizes the fourth-order cumulant to construct the rotation invariance structure and then combine the ESPRIT algorithm to estimate the DOA and polarization information of the incident signals based on uniform circular EVSAs. The proposed algorithm utilizes only part of the fourth-order cumulant matrix which contains non-redundant information to reduce the computational cost, other than the whole matrix.

The rest of the paper is organized as follows: the uniform circular array composed of orthogonal dipoles and its signal model are described in Section 2. The virtual array extension of the uniform circular EVSA consisting of orthogonal dipoles is introduced in Section 3. Based on the virtual extended array constructed from fourth-order cumulant in Section 3, the method of the rotation invariance structure construction and joint estimation of DOA and polarization information of the incident signals, is presented in details in Section 4. In Section 5, the effectiveness of the proposed algorithm is demonstrated by numerical examples. The conclusions and possible research expectations for future work are outlined in Section 6. Regarding the notations used in this paper, the operator ⊗ denotes the Kronecker product; angle(⋅) denotes the phase of a complex number; E{⋅} denotes the expected value; diag(⋅) denotes a diagonal matrix composed of the columns or row vectors; cum(⋅) denotes the fourth-order cumulants; ${\left(\cdot \right)}^{*}$, ${\left(\cdot \right)}^{T}$, ${\left(\cdot \right)}^{H}$ and ${\left(\cdot \right)}^{+}$ represent the complex conjugate, transpose, conjugate transpose and matrix inverse, respectively.

## 2. Array Signal Model

**Figure 1:**
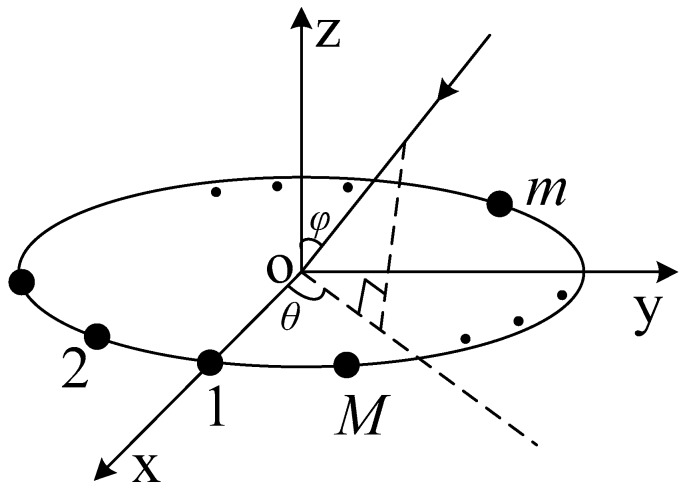
Structure of the uniform circular EVSA.

As shown in Figure 1, K non-Gaussian, narrowband, far-field, incoherent plane wave signals imping on a uniform circular array with M array elements, which are uniformly distributed along a circular path with the radius of r. The element located on the positive x axis is denoted as “1”; the remaining elements are uniformly arranged clockwise on the path, successively denoted as “2” to “*M*”. The phase center of each element is always located on the xoy-plane. Each element consists of two dipoles that are spatially co-located and orthogonal to each other, leading to the array composed of N=2M dipoles.

In Figure 1, θ∈[0,2π] and φ∈[0,π/2] are the azimuth angle and elevation angle of the incident signal, respectively. Here we introduce another two angles, γ∈[0,π/2] and η∈[0,2π], referred to as the polarization auxiliary angle and polarization phase difference, respectively, to completely describe the polarization state of the incident signal.

Here, assuming that the noise is the additive white Gaussian noise and independent of the incident signals. In real direction finding system, in order to facilitate processing, amplitudes of received data are always compensated and the mutual coupling between array elements [47,48] are calibrated before they are provided to direction finding signal processor, so that the received data of each element is regarded as uniform in term of amplitude and effect of the mutual coupling. Therefore, the output of array can be expressed as:

(1) $$x(t)=\sum_{k=1}^{K}a(\theta_{k},\varphi_{k},\gamma_{k},\eta_{k})s_{k}(t)+n(t)=As(t)+n(t)$$

where x(t) is an N×1 received signal vector, s(t) is a K×1 incident signal vector, n(t) is an N×1 Gaussian white noise vector with zero mean and noise power $\sigma_{N}^{2}$, and A is the N×K array manifold matrix formed by the set of the K array manifold vectors $a(\theta_{k},\varphi_{k},\gamma_{k},\eta_{k})$ which can be expressed as:

(2) $$a(\theta_{k,}\varphi_{k},\gamma_{k},\eta_{k})=a_{S}(\theta_{k,}\varphi_{k})⊗a_{P}(\theta_{k,}\varphi_{k},\gamma_{k},\eta_{k})$$

where $a_{S}(\theta_{k,}\varphi_{k})$ is the spatial steering vector and $a_{P}(\theta_{k,}\varphi_{k},\gamma_{k},\eta_{k})$ is the polarization-spatial domain steering vector. The expressions of the two vectors are shown as follows:

(3) $$a_{S}(\theta_{k,}\varphi_{k})={\left[e^{-j2\pi (r\sin\varphi_{k}\cos(-\theta_{k}))/\lambda_{k}},\cdots ,e^{-j2\pi (r\sin\varphi_{k}\cos(\frac{2\pi (m-1)}{M}-\theta_{k}))/\lambda_{k}},\cdots ,e^{-j2\pi (r\sin\varphi_{k}\cos(\frac{2\pi (M-1)}{M}-\theta_{k}))/\lambda_{k}}\right]}^{T}$$

(4) $$a_{P}(\theta_{k,}\varphi_{k},\gamma_{k},\eta_{k})=\left[\begin{array}{cc} -\sin\theta_{k} & \cos\varphi_{k}\cos\theta_{k}\\ \cos\theta_{k} & \cos\varphi_{k}\sin\theta_{k} \end{array}\right]\left[\begin{array}{c} \cos\gamma_{k}\\ \sin\gamma_{k}e^{j\eta_{k}} \end{array}\right]$$

According to x(t), the observed output of identical polarization directions can then be obtained as:

(5) $$\{\begin{array}{c} x_{x}(t)=I_{M}⊗e_{x}^{T}x(t)=A_{x}s(t)+n_{x}(t)\\ x_{y}(t)=I_{M}⊗e_{y}^{T}x(t)=A_{y}s(t)+n_{y}(t) \end{array}$$

where $I_{M}$ is an M×M identity matrix, $e_{x}={\left[\begin{array}{cc} 1 & 0 \end{array}\right]}^{T}$, $e_{y}={\left[\begin{array}{cc} 0 & 1 \end{array}\right]}^{T}$, $A_{x}=I_{M}⊗e_{x}^{T}A$, $A_{y}=I_{M}⊗e_{y}^{T}A$, $n_{x}(t)=I_{M}⊗e_{x}^{T}n(t)$ and $n_{y}(t)=I_{M}⊗e_{y}^{T}n(t)$, so that $A_{x}$ and $A_{y}$ are respectively formed by the set of the K array steering vectors $a_{x}(\theta_{k},\varphi_{k},\gamma_{k},\eta_{k})$ and $a_{y}(\theta_{k},\varphi_{k},\gamma_{k},\eta_{k})$, which can be expressed as:

(6) $$a_{x}(\theta_{k},\varphi_{k},\gamma_{k},\eta_{k})=\left[\begin{array}{cc} -\sin\theta_{k} & \cos\varphi_{k}\cos\theta_{k} \end{array}\right]\left[\begin{array}{c} \cos\gamma_{k}\\ \sin\gamma_{k}e^{j\eta_{k}} \end{array}\right]a_{S}(\theta_{k,}\varphi_{k})=P_{x}(\theta_{k},\varphi_{k},\gamma_{k},\eta_{k})a_{S}(\theta_{k,}\varphi_{k})$$

(7) $$a_{y}(\theta_{k},\varphi_{k},\gamma_{k},\eta_{k})=\left[\begin{array}{cc} \cos\theta_{k} & \cos\varphi_{k}\sin\theta_{k} \end{array}\right]\left[\begin{array}{c} \cos\gamma_{k}\\ \sin\gamma_{k}e^{j\eta_{k}} \end{array}\right]a_{S}(\theta_{k,}\varphi_{k})=P_{y}(\theta_{k},\varphi_{k},\gamma_{k},\eta_{k})a_{S}(\theta_{k,}\varphi_{k})$$

where $P_{x}(\theta_{k},\varphi_{k},\gamma_{k},\eta_{k})$ and $P_{y}(\theta_{k},\varphi_{k},\gamma_{k},\eta_{k})$ are scalars and denote the two components of polarization-spatial domain steering vector with the polarization direction along the x-axis and y-axis, respectively. For any incident signals, $P_{x}(\theta_{k},\varphi_{k},\gamma_{k},\eta_{k})$ and $P_{y}(\theta_{k},\varphi_{k},\gamma_{k},\eta_{k})$ are two different complex numbers. The module value of them can be regarded as gains of two dipoles generated by polarization reception, respectively, and the phase angles of them can be regarded as additional phase differences of two dipoles generated by polarization receiving, respectively.

Then, a renewed received signal vector can be obtained as:

(8) $$\tilde{x}(t)=e_{x}⊗x_{x}(t)+e_{y}⊗x_{y}(t)=\tilde{A}s(t)+\tilde{n}(t)$$

where $\tilde{A}=e_{x}⊗A_{x}(t)+e_{y}⊗A_{y}(t)$, $\tilde{n}(t)=e_{x}⊗n_{x}(t)+e_{y}⊗n_{y}(t)$. $\tilde{A}$ is formed by the set of the K array steering vectors ${\tilde{a}}_{k}(\theta_{k},\varphi_{k},\gamma_{k},\eta_{k})$, which can be expressed as:

(9) $$\begin{array}{cc} {\tilde{a}}_{k}(\theta_{k},\varphi_{k},\gamma_{k},\eta_{k}) & =e_{x}⊗I_{M}⊗e_{x}^{T}a(\theta_{k},\varphi_{k},\gamma_{k},\eta_{k})+e_{y}⊗I_{M}⊗e_{y}^{T}a(\theta_{k},\varphi_{k},\gamma_{k},\eta_{k})\\ & =e_{x}⊗(P_{x}(\theta_{k},\varphi_{k},\gamma_{k},\eta_{k})a_{S}(\theta_{k},\varphi_{k}))+e_{y}⊗(P_{y}(\theta_{k},\varphi_{k},\gamma_{k},\eta_{k})a_{S}(\theta_{k},\varphi_{k})) \end{array}$$

## 3. Virtual Array Extension of the Uniform Circular EVSA Consisting of Orthogonal Dipoles

Assuming that the signal is a zero-mean, non-Gaussian, stationary random process, the fourth-order cumulant can be defined as:

(10) $$\begin{array}{cc} C_{4x}(k_{1},k_{2},k_{3},k_{4}) & =cum\{x_{k_{1}},x_{k_{2}},x_{k_{3}}^{*},x_{k_{4}}^{*}\}\\ & =E\{x_{k_{1}}x_{k_{2}}x_{k_{3}}^{*}x_{k_{4}}^{*}\}-E\{x_{k_{1}}x_{k_{3}}^{*}\}E\{x_{k_{2}}x_{k_{4}}^{*}\}\\ & -E\{x_{k_{1}}x_{k_{4}}^{*}\}E\{x_{k_{2}}x_{k_{3}}^{*}\}-E\{x_{k_{1}}x_{k_{2}}\}E\{x_{k_{3}}^{*}x_{k_{4}}^{*}\} \end{array}$$

The results of Equation (10) can be collected in the form of matrixand denoted by the cumulant matrix $R_{4}$ as:

(11) $$R_{4}((k_{1}-1)N+k_{2},(k_{3}-1)N+k_{4})=C_{4x}(k_{1},k_{2},k_{3},k_{4})$$

where $R_{4}$ is the fourth-order cumulant matrix. If the incident signals are independent of each other, $R_{4}$ can be written as:

(12) $$\begin{array}{cc} R_{4} & =E\{(x⊗x){\left(x⊗x\right)}^{H}\}-E\{(x⊗x)\}E\{{\left(x⊗x\right)}^{H}\}\\ & -E\{(xx^{H})\}⊗E\{(xx^{H})\}\\ & =B(\theta ,\varphi ,\gamma ,\eta )C_{S}B^{H}(\theta ,\varphi ,\gamma ,\eta ) \end{array}$$

where $C_{S}=\mathrm{diag}(\gamma_{4s_{k}})$, and the steering vector matrix B(θ,φ,γ,η) is expressed in the following form:

(13) $$\begin{array}{cc} B(\theta ,\varphi ,\gamma ,\eta )= & \left[\begin{array}{ccc} b(\theta_{1},\varphi_{1},\gamma_{1},\eta_{1}) & \cdots & b(\theta_{K},\varphi_{K},\gamma_{K},\eta_{K}) \end{array}\right]\\ & =[\begin{array}{ccc} \tilde{a}(\theta_{1},\varphi_{1},\gamma_{1},\eta_{1})⊗\tilde{a}(\theta_{1},\varphi_{1},\gamma_{1},\eta_{1}) & \cdots & \tilde{a}(\theta_{K},\varphi_{K},\gamma_{K},\eta_{K})⊗\tilde{a}(\theta_{K},\varphi_{K},\gamma_{K},\eta_{K}) \end{array}] \end{array}$$

Based on the equations above, it can be inferred that the fourth-order cumulant matrix of the received data can be considered as the covariance matrix of the data received by a virtual extended array, and the corresponding array steering vector of the *k*th signal can be expressed as follows:

(14) $$b(\theta_{k},\varphi_{k},\gamma_{k},\eta_{k})=\tilde{a}(\theta_{k},\varphi_{k},\gamma_{k},\eta_{k})⊗\tilde{a}(\theta_{k},\varphi_{k},\gamma_{k},\eta_{k})$$

The coefficients of the virtual array steering vector can be regarded as the gains and additional phase differences of the elements in the virtual extended array. By separating the elements of the array steering vector according to different coefficient from vector $b(\theta_{k},\varphi_{k},\gamma_{k},\eta_{k})$, four vectors can be obtained as follows:

(15) $$\begin{array}{l} c_{xx}(\theta_{k},\varphi_{k},\gamma_{k},\eta_{k})=(I_{M}⊗e_{x}^{T}⊗I_{M})(e_{x}^{T}⊗I_{2M^{2}})b(\theta_{k},\varphi_{k},\gamma_{k},\eta_{k})\\ \;=P_{x}^{2}(\theta_{k},\varphi_{k},\gamma_{k},\eta_{k})a_{S}(\theta_{k},\varphi_{k})⊗a_{S}(\theta_{k},\varphi_{k}) \end{array}$$

(16) $$\begin{array}{l} c_{xy}(\theta_{k},\varphi_{k},\gamma_{k},\eta_{k})=(I_{M}⊗e_{y}^{T}⊗I_{M})(e_{y}^{T}⊗I_{2M^{2}})b(\theta_{k},\varphi_{k},\gamma_{k},\eta_{k})\\ \;=P_{x}(\theta_{k},\varphi_{k},\gamma_{k},\eta_{k})P_{y}(\theta_{k},\varphi_{k},\gamma_{k},\eta_{k})a_{S}(\theta_{k},\varphi_{k})⊗a_{S}(\theta_{k},\varphi_{k}) \end{array}$$

(17) $$\begin{array}{l} c_{yx}(\theta_{k},\varphi_{k},\gamma_{k},\eta_{k})=(I_{M}⊗e_{x}^{T}⊗I_{M})(e_{y}^{T}⊗I_{2M^{2}})b(\theta_{k},\varphi_{k},\gamma_{k},\eta_{k})\\ \;=P_{y}(\theta_{k},\varphi_{k},\gamma_{k},\eta_{k})P_{x}(\theta_{k},\varphi_{k},\gamma_{k},\eta_{k})a_{S}(\theta_{k},\varphi_{k})⊗a_{S}(\theta_{k},\varphi_{k}) \end{array}$$

(18) $$\begin{array}{l} c_{yy}(\theta_{k},\varphi_{k},\gamma_{k},\eta_{k})=(I_{M}⊗e_{y}^{T}⊗I_{M})(e_{y}^{T}⊗I_{2M^{2}})b(\theta_{k},\varphi_{k},\gamma_{k},\eta_{k})\\ \;=P_{y}^{2}(\theta_{k},\varphi_{k},\gamma_{k},\eta_{k})a_{S}(\theta_{k},\varphi_{k})⊗a_{S}(\theta_{k},\varphi_{k}) \end{array}$$

where $P_{x}^{2}(\theta ,\varphi ,\gamma ,\eta )$, $P_{x}(\theta ,\varphi ,\gamma ,\eta )P_{y}(\theta ,\varphi ,\gamma ,\eta )$, $P_{y}(\theta ,\varphi ,\gamma ,\eta )P_{x}(\theta ,\varphi ,\gamma ,\eta )$ and $P_{y}^{2}(\theta ,\varphi ,\gamma ,\eta )$ are the coefficients of the virtual array steering vector, respectively.

Separating the rows of the fourth-order cumulant matrix $R_{4}$, which follows the same order of Equations (15)–(18), we obtain:

(19) $$R_{4xx}=(I_{M}⊗e_{x}^{T}⊗I_{M})(e_{x}^{T}⊗I_{2M^{2}})R_{4}\;=c_{xx}(\theta ,\varphi ,\gamma ,\eta )C_{S}B^{H}(\theta ,\varphi ,\gamma ,\eta )$$

(20) $$R_{4xy}=(I_{M}⊗e_{y}^{T}⊗I_{M})(e_{x}^{T}⊗I_{2M^{2}})R_{4}=c_{xy}(\theta ,\varphi ,\gamma ,\eta )C_{S}B^{H}(\theta ,\varphi ,\gamma ,\eta )$$

(21) $$R_{4yx}=(I_{M}⊗e_{x}^{T}⊗I_{M})(e_{y}^{T}⊗I_{2M^{2}})R_{4}\;=c_{yx}(\theta ,\varphi ,\gamma ,\eta )C_{S}B^{H}(\theta ,\varphi ,\gamma ,\eta )$$

(22) $$R_{4yy}=(I_{M}⊗e_{y}^{T}⊗I_{M})(e_{y}^{T}⊗I_{2M^{2}})R_{4}=c_{yy}(\theta ,\varphi ,\gamma ,\eta )C_{S}B^{H}(\theta ,\varphi ,\gamma ,\eta )$$

Then, a new fourth-order cumulant matrix ${\overset{⌢}{R}}_{4}$ can be expressed as:

(23) $$\begin{array}{ll} {\overset{⌢}{R}}_{4} & =e_{x}⊗e_{x}⊗R_{4xx}+e_{x}⊗e_{y}⊗R_{4xy}+e_{y}⊗e_{x}⊗R_{4yx}+e_{y}⊗e_{y}⊗R_{4yy}\\ & =\overset{⌢}{B}(\theta ,\varphi ,\gamma ,\eta )C_{S}B^{H}(\theta ,\varphi ,\gamma ,\eta ) \end{array}$$

(24) $$\overset{⌢}{B}(\theta ,\varphi ,\gamma ,\eta )=[\begin{array}{ccc} c(\theta_{1},\varphi_{1},\gamma_{1},\eta_{1}) & \cdots & c(\theta_{K},\varphi_{K},\gamma_{K},\eta_{K}) \end{array}]$$

(25) $$c(\theta_{k},\varphi_{k},\gamma_{k},\eta_{k})=c_{P}(\theta_{k},\varphi_{k},\gamma_{k},\eta_{k})⊗c_{S}(\theta_{k},\varphi_{k})$$

where, $c_{S}(\theta_{k},\varphi_{k})=a_{S}(\theta_{k},\varphi_{k})⊗a_{S}(\theta_{k},\varphi_{k})$ is the spatial steering vector of the virtual array and then the polarization-spatial domain steering vector $c_{P}(\theta_{k},\varphi_{k},\gamma_{k},\eta_{k})$ can be written as:

(26) $$c_{P}(\theta_{k},\varphi_{k},\gamma_{k},\eta_{k})=\left[\begin{array}{cccc} \sin^{2}\theta_{k} & -\sin\theta_{k}\cos\theta_{k}\cos\varphi_{k} & -\sin\theta_{k}\cos\theta_{k}\cos\varphi_{k} & \cos^{2}\theta_{k}\cos^{2}\varphi_{k}\\ -\sin\theta_{k}\cos\theta_{k} & \cos^{2}\theta_{k}\cos\varphi_{k} & -\sin^{2}\theta_{k}\cos\varphi_{k} & \sin\theta_{k}\cos\theta_{k}\cos^{2}\varphi_{k}\\ -\sin\theta_{k}\cos\theta_{k} & \cos^{2}\theta_{k}\cos\varphi_{k} & -\sin^{2}\theta_{k}\cos\varphi_{k} & \sin\theta_{k}\cos\theta_{k}\cos^{2}\varphi_{k}\\ \cos^{2}\theta_{k} & \sin\theta_{k}\cos\theta_{k}\cos\varphi_{k} & \sin\theta_{k}\cos\theta_{k}\cos\varphi_{k} & \sin^{2}\theta_{k}\cos^{2}\varphi_{k} \end{array}\right]\left[\begin{array}{c} \cos^{2}\gamma \\ \sin\gamma \cos\gamma e^{j\eta }\\ \sin\gamma \cos\gamma e^{j\eta }\\ \sin^{2}\gamma e^{j2\eta } \end{array}\right]$$

According to the Equations (25) and (26), it can be observed that $R_{4xy}$ and $R_{4yx}$ have identical expressions, thus both of them can be regarded as the output of the same dipole. Therefore, each element of the virtual array consists of three spatially co-located dipoles, and the polarization-spatial domain steering vector of the virtual array has three components ($P_{x}^{2}(\theta ,\varphi ,\gamma ,\eta )$, $P_{y}(\theta ,\varphi ,\gamma ,\eta )P_{x}(\theta ,\varphi ,\gamma ,\eta )$ and $P_{y}^{2}(\theta ,\varphi ,\gamma ,\eta )$) along the corresponding polarization direction.

**Figure 2:**
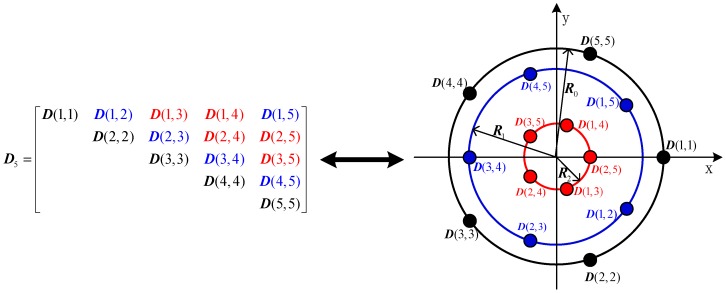
Matrix ***D***_5_ and the corresponding virtual array.

**Figure 3:**
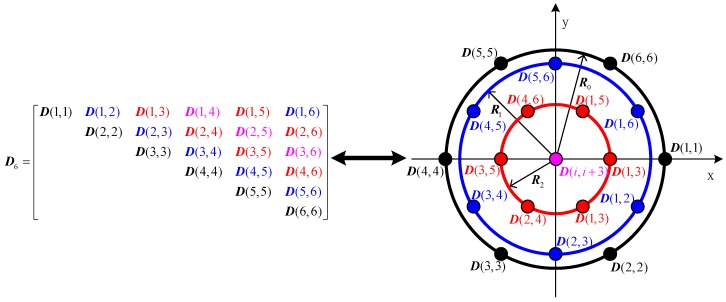
Matrix ***D***_6_ and the corresponding virtual array.

The spatial steering vector of the virtual array $c_{S}(\theta_{k},\varphi_{k})$ can be rewritten in the following matrix form:

(27) $$D=\left[\begin{array}{cccc} a_{S1}(\theta_{k},\varphi_{k})a_{S1}(\theta_{k},\varphi_{k}) & a_{S2}(\theta_{k},\varphi_{k})a_{S1}(\theta_{k},\varphi_{k}) & \cdots & a_{SM}(\theta_{k},\varphi_{k})a_{S1}(\theta_{k},\varphi_{k})\\ a_{S1}(\theta_{k},\varphi_{k})a_{S2}(\theta_{k},\varphi_{k}) & a_{S2}(\theta_{k},\varphi_{k})a_{S2}(\theta_{k},\varphi_{k}) & \cdots & a_{SM}(\theta_{k},\varphi_{k})a_{S2}(\theta_{k},\varphi_{k})\\ \vdots & \vdots & \ddots & \vdots \\ a_{S1}(\theta_{k},\varphi_{k})a_{SM}(\theta_{k},\varphi_{k}) & a_{S2}(\theta_{k},\varphi_{k})a_{SM}(\theta_{k},\varphi_{k}) & \cdots & a_{SM}(\theta_{k},\varphi_{k})a_{SM}(\theta_{k},\varphi_{k}) \end{array}\right]$$

where $D(i,j)=c_{S}((i-1)M+j)$, i.e., the (i−1)M+jth element of $c_{S}(\theta_{k},\varphi_{k})$ as the *i*th row and *j*th column of matrix D. Based on the above derivation, the characteristics of the virtual extended array are summarized as follows:

1. When the array element number is odd (M=2n+1), the element number of the virtual extended array is $2n^{2}+3n+1$, where each element consists of three co-located dipoles. The virtual array consists of n+1 concentric uniform circular arrays with the same element number of 2n+1. The elements of the virtual array corresponding to the elements on the main diagonal of matrix D, constitute the largest uniform circular array with the radius of 2r, and the elements located on the i-th and M−i-th diagonals which are parallel to the main diagonal of matrix D, constitute a uniform circular array with the radius of 2r|cos(πi/M)|.
  For example, the matrix $D_{5}$ and the virtual array of uniform circular array consisting of five elements are illustrated in Figure 2. D(i,j) is equal to D(j,i), so that only the 15 upper triangular elements in matrix $D_{5}$ are shown (Figure 2, left). The right part of Figure 2 shows the virtual array which is equivalent to the matrix $D_{5}$. To show the relation of $D_{5}$ to the virtual array, the elements of $D_{5}$ and the corresponding antenna elements of the virtual array are marked with the same color. The virtual array is composed of three concentric uniform circular arrays, each of which consists of five elements. The radii of the uniform circular arrays are $R_{0}=2r$, $R_{1}=2r\left|\cos(36^{\circ })\right|$ and $R_{2}=2r\left|\cos(72^{\circ })\right|$, respectively.
2. When the array element number is even (M=2n), the element number of the virtual extended array is $2n^{2}+1$, where each element consists of three co-located dipoles. The virtual array consists of one element located at the origin of the coordinate system and n concentric uniform circular arrays with the same element number of 2n. The elements of virtual array corresponding to the elements on the main diagonal of matrix D, constitute the largest uniform circular array of radius 2r, and the elements located on the i-th and M−i-th diagonal which are parallel to the main diagonal of matrix D, constitute a uniform circular array of radius 2r|cos(πi/M)|. The elements located on the n+1-th diagonal of matrix D correspond to the virtual elements located at the origin of the coordinate system. In order to express this situation more clearly, an example of a uniform circular array composed of 6 elements is illustrated in Figure 3, where the matrix $D_{6}$ (left) and the corresponding virtual array (right) are shown.

As before, the corresponding elements of ***D***_6_ and the elements of the virtual array have the same color. The virtual array is comprised of one element located at the origin of the coordinate system and three concentric uniform circular arrays. Each uniform circular array has six elements. The radii of the uniform circular arrays are $R_{0}=2r$, $R_{1}=2r\left|\cos(30^{\circ })\right|$ and $R_{2}=r$, respectively.

It can be inferred that the cross-dipole uniform circular array can be extended to a set of uniform circular arrays (or a set of uniform circular arrays with one additional element located at the origin of the coordinate system), whose elements consist of three co-located dipoles, by constructing the fourth-order cumulant of the received data. Thus, some pairs of sub-arrays with rotational-invariance can be obtained, and then the DOA and polarization of the incident signals can be jointly estimated based on the ESPRIT algorithm.

## 4. Proposed Algorithm

**Figure 4:**
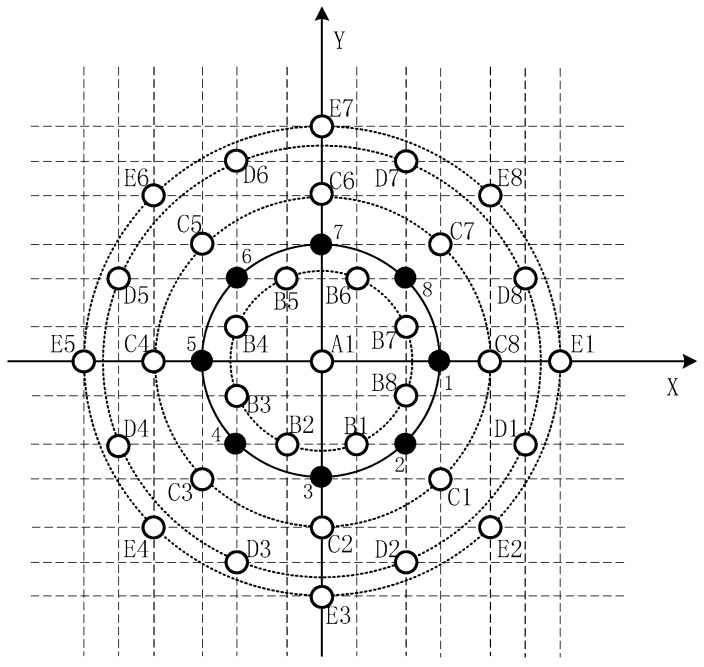
The corresponding virtual extension array of the uniform circular EVSA with eight elements.

For the purposes of discussion, a uniform circular array equipped with eight elements is analyzed as an example in this section. However, the concepts presented in this section are also applicable to a uniform circular array with arbitrary element number. The corresponding virtual extended array is drawn as a diagram as shown in Figure 4.

In Figure 4, the black elements represent the actual array and the white ones represent the virtual arrays derived from the actual array. The element located at the origin of the Cartesian coordinate system is labeled as A1, and the other elements located on four concentric uniform circular paths are labeled as Bi, Ci, Di and Ei (i=1,2,⋯,8), where the array elements B1, C1, D1 and E1 correspond to the elements D(1,4), D(1,3), D(1,2) and D(1,1) of the matrix D, respectively.

### 4.1. Selection of Rotational Invariant Sub-Array Pairs

In order to avoid peak searching and reduce the computational complexity, we develop an ESPRIT algorithm based on the fourth-order cumulant to exploit the property of rotational invariance. It is therefore necessary to search for those pairs of sub-arrays that are provided with rotational invariance. In order to estimate the two-dimensional DOAs, at least two pairs of sub-arrays are required. The idea behind searching for sub-array pairs available for DOA and polarization estimation is proposed as follows.

First, the spatial phase factors between the virtual elements A1 and B1, A1 and B3 are defined as the rotation invariant factors, which can be expressed as:

(28) $$\begin{array}{l} u_{B1}=a_{S4}(\theta ,\varphi )a_{S1}(\theta ,\varphi )\\ \;=\exp(-j2\pi ((d_{o4}+d_{o1})\vec{r})/\lambda )\\ \;=\exp(-j2\pi r((1-\sqrt{2}/2)\sin\varphi \cos\theta +\sqrt{2}/2\sin\varphi \sin\theta )/\lambda ) \end{array}$$

(29) $$\begin{array}{l} u_{B3}=a_{S6}(\theta ,\varphi )a_{S3}(\theta ,\varphi )\\ \;=\exp(-j2\pi ((d_{o6}+d_{o3})\vec{r})/\lambda )\\ \;=\exp(-j2\pi r(-\sqrt{2}/2\sin\varphi \cos\theta +(1-\sqrt{2}/2)\sin\varphi \sin\theta )/\lambda ) \end{array}$$

Based on these two rotation invariant factors, two pairs of sub-arrays with the property of rotational invariance can be obtained as:

**Pair** **1:** Sub-array 1:A1, B2, B5, B8, C3, C5, C6, C8, D4, D5, D7, D8, E5, E8
  Sub-array 2:B1, C2, A1, C1, D3, B4, B6, D1, E4, C4, C7, E1, D4, D8
**Pair** **2:** Sub-array 3:A1, B2, B4, B7, C2, C5, C7, C8, D1, D2, D6, D7, E2, E7
  Sub-array 4:B3, C3, C4, A1, D3, D5, B6, B8, C1, E3, E6, C6, D2, D6

**Figure 5:**
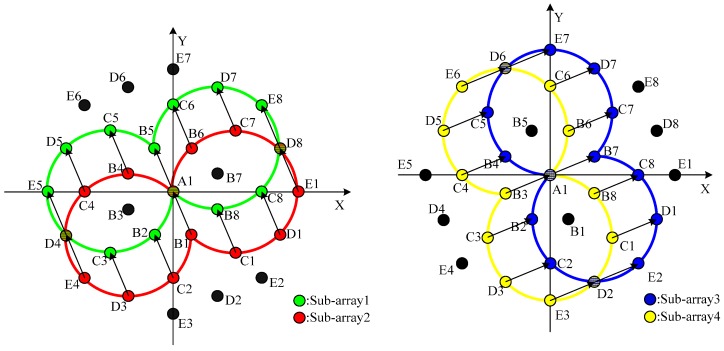
Two pairs of sub-arrays with the property of rotational invariance.

In order to display the pairs of sub-arrays clearly, the four sub-arrays (Sub-array 1, 2, 3 and 4) are drawn in four different colors, as shown in Figure 5. It is obvious that Sub-array 1 and Sub-array 2 have the property of rotational invariance, and $u_{B1}$ is the rotation invariant factor. It’s the same case for Sub-array 3 and Sub-array 4, where $u_{B3}$ is the rotation invariant factor.

It can be observed that all of the elements in the virtual array are contained within the two pairs of sub-arrays shown above. As each element consists of three virtual dipoles, the algorithm efficiently takes advantages of all non-redundant spatial information of the fourth-order cumulant matrices. The DOA estimation can be accomplished by using one of the three groups of dipoles, which has the same gain and additional phase difference. In general, to improve the estimation accuracy, the algorithm usually uses more dipoles consequently resulting in higher computation complexity. In order to reduce the computational complexity effectively, dipoles of one group are only used in the proposed algorithm while ensuring the estimation accuracy.

In addition, any two elements of Bi separated by one array element can be selected as the spatial phase factor, and the two corresponding pairs of sub-arrays then can be used to realize two-dimensional DOA estimation.

### 4.2. Two-Dimensional DOA Estimation

The relationships between the elements of the virtual sub-arrays and the elements of matrix D are listed in Table 1.

Based on the equation $D(i,j)=c_{S}((i-1)M+j)$, and the corresponding relationships between the elements of the virtual extended array and the rows of matrix ${\overset{⌢}{R}}_{4}$, the corresponding selection matrices $E_{1}$, $E_{2}$, $E_{3}$ and $E_{4}$ can be constructed as follows:

(30) $$E_{1}={\left[\begin{array}{cccc} e_{(i_{sub11}-1)M+j_{sub11}} & e_{(i_{sub12}-1)M+j_{sub12}} & \cdots & e_{(i_{sub1N^{\prime }}-1)M+j_{sub1N^{\prime }}} \end{array}\right]}^{T}$$

(31) $$E_{2}={\left[\begin{array}{cccc} e_{(i_{sub21}-1)M+j_{sub21}} & e_{(i_{sub22}-1)M+j_{sub22}} & \cdots & e_{(i_{sub2N^{\prime }}-1)M+j_{sub2N^{\prime }}} \end{array}\right]}^{T}$$

(32) $$E_{3}={\left[\begin{array}{cccc} e_{(i_{sub31}-1)M+j_{sub31}} & e_{(i_{sub32}-1)M+j_{sub32}} & \cdots & e_{(i_{sub3N^{\prime }}-1)M+j_{sub3N^{\prime }}} \end{array}\right]}^{T}$$

(33) $$E_{4}={\left[\begin{array}{cccc} e_{(i_{sub41}-1)M+j_{sub41}} & e_{(i_{sub42}-1)M+j_{sub42}} & \cdots & e_{(i_{sub4N^{\prime }}-1)M+j_{sub4N^{\prime }}} \end{array}\right]}^{T}$$

where the matrix element corresponding to the *n*-th element of the m-th sub-array appears as the $i_{submn}$-th row and $j_{submn}$-th column of matrix D. $N^{\prime }$ denotes the total number of sub-arrays, which is 14 for the eight-sensor array. $e_{(i_{submn}-1)M+j_{submn}}$ denotes a $M^{2}\times 1$ vector with its $\left((i_{submn}-1)M+j_{submn}\right)$th element as the only non-zero item.

Therefore, we can get:

(34) $$R_{4xx1}=E_{1}R_{4xx}=B_{1}(\theta ,\varphi ,\gamma ,\eta )C_{S}B^{H}(\theta ,\varphi ,\gamma ,\eta )$$

(35) $$R_{4xx2}=E_{2}R_{4xx}=B_{2}(\theta ,\varphi ,\gamma ,\eta )C_{S}B^{H}(\theta ,\varphi ,\gamma ,\eta )$$

(36) $$R_{4xx3}=E_{3}R_{4xx}=B_{3}(\theta ,\varphi ,\gamma ,\eta )C_{S}B^{H}(\theta ,\varphi ,\gamma ,\eta )$$

(37) $$R_{4xx4}=E_{4}R_{4xx}=B_{4}(\theta ,\varphi ,\gamma ,\eta )C_{S}B^{H}(\theta ,\varphi ,\gamma ,\eta )$$

where $B_{1}(\theta ,\varphi ,\gamma ,\eta )$, $B_{2}(\theta ,\varphi ,\gamma ,\eta )$, $B_{3}(\theta ,\varphi ,\gamma ,\eta )$ and $B_{4}(\theta ,\varphi ,\gamma ,\eta )$ denote the steering vectors of sub-array 1, sub-array 2, sub-array 3 and sub-array 4, respectively. The relationships between $B_{1}(\theta ,\varphi ,\gamma ,\eta )$, $B_{2}(\theta ,\varphi ,\gamma ,\eta )$, $B_{3}(\theta ,\varphi ,\gamma ,\eta )$ and $B_{4}(\theta ,\varphi ,\gamma ,\eta )$ can be expressed as follows:

(38) $$B_{2}(\theta ,\varphi ,\gamma ,\eta )=B_{1}(\theta ,\varphi ,\gamma ,\eta )\Phi_{1}$$

(39) $$B_{4}(\theta ,\varphi ,\gamma ,\eta )=B_{3}(\theta ,\varphi ,\gamma ,\eta )\Phi_{2}$$

where $\Phi_{1}=\mathrm{diag}(u_{B1}(\theta_{1},\varphi_{1},\gamma_{1},\eta_{1}),u_{B1}(\theta_{2},\varphi_{2},\gamma_{2},\eta_{2}),\cdots ,u_{B1}(\theta_{K},\varphi_{K},\gamma_{K},\eta_{K}))$, $\Phi_{2}=\mathrm{diag}(u_{B3}(\theta_{1},\varphi_{1},\gamma_{1},\eta_{1}),u_{B3}(\theta_{2},\varphi_{2},\gamma_{2},\eta_{2}),\cdots ,u_{B3}(\theta_{K},\varphi_{K},\gamma_{K},\eta_{K}))$.

Furthermore, we have:

(40) $$\{\begin{array}{l} R_{4xx1}=B_{1}(\theta ,\varphi ,\gamma ,\eta )C_{S}B^{H}(\theta ,\varphi ,\gamma ,\eta )\\ R_{4xx2}=B_{1}(\theta ,\varphi ,\gamma ,\eta )\Phi_{1}C_{S}B^{H}(\theta ,\varphi ,\gamma ,\eta ) \end{array}$$

(41) $$\{\begin{array}{l} R_{4xx3}=B_{3}(\theta ,\varphi ,\gamma ,\eta )C_{S}B^{H}(\theta ,\varphi ,\gamma ,\eta )\\ R_{4xx4}=B_{3}(\theta ,\varphi ,\gamma ,\eta )\Phi_{2}C_{S}B^{H}(\theta ,\varphi ,\gamma ,\eta ) \end{array}$$

Then, matrices $C_{1}$ and $C_{2}$ are constructed based on Equations (40) and (41) as:

(42) $$C_{1}=\left[\begin{array}{c} R_{4xx1}\\ R_{4xx2} \end{array}\right]=\left[\begin{array}{c} B_{1}(\theta ,\varphi ,\gamma ,\eta )\\ B_{1}(\theta ,\varphi ,\gamma ,\eta )\Phi_{1} \end{array}\right]C_{S}B^{H}(\theta ,\varphi ,\gamma ,\eta )$$

(43) $$C_{2}=\left[\begin{array}{c} R_{4xx3}\\ R_{4xx4} \end{array}\right]=\left[\begin{array}{c} B_{3}(\theta ,\varphi ,\gamma ,\eta )\\ B_{3}(\theta ,\varphi ,\gamma ,\eta )\Phi_{2} \end{array}\right]C_{S}B^{H}(\theta ,\varphi ,\gamma ,\eta )$$

When using singular value decomposition (SVD), $C_{1}$ and $C_{2}$ can be denoted as:

(44) $$C_{1}=\left[\begin{array}{cc} U_{S1} & U_{N1} \end{array}\right]\left[\begin{array}{cc} \Sigma_{S1} & \\ & \Sigma_{N1} \end{array}\right]\left[\begin{array}{c} V_{S1}^{H}\\ V_{N1}^{H} \end{array}\right]$$

(45) $$C_{2}=\left[\begin{array}{cc} U_{S2} & U_{N2} \end{array}\right]\left[\begin{array}{cc} \Sigma_{S2} & \\ & \Sigma_{N2} \end{array}\right]\left[\begin{array}{c} V_{S2}^{H}\\ V_{N2}^{H} \end{array}\right]$$

The subspace spanned by array steering vector is included in the signal subspace, hence there exists a nonsingular matrix T expressed as:

(46) $$\left[\begin{array}{c} B_{1}(\theta ,\varphi ,\gamma ,\eta )\\ B_{1}(\theta ,\varphi ,\gamma ,\eta )\Phi_{1} \end{array}\right]T_{1}=U_{S1}=\left[\begin{array}{c} U_{S1_{1}}\\ U_{S1_{2}} \end{array}\right]$$

(47) $$\left[\begin{array}{c} B_{3}(\theta ,\varphi ,\gamma ,\eta )\\ B_{3}(\theta ,\varphi ,\gamma ,\eta )\Phi_{2} \end{array}\right]T_{2}=U_{S2}=\left[\begin{array}{c} U_{S2_{1}}\\ U_{S2_{2}} \end{array}\right]$$

Consequently, we have:

(48) $$U_{S1_{2}}=U_{S1_{1}}T_{1}^{-1}\Phi_{1}T_{1}=U_{S1_{1}}\Psi_{1}$$

(49) $$U_{S2_{2}}=U_{S2_{1}}T_{2}^{-1}\Phi_{2}T_{2}=U_{S2_{1}}\Psi_{2}$$

so, the total least square solutions of Equations (48) and (49) are:

(50) $$\Psi_{1}=U_{S1_{1}}^{+}U_{S1_{2}}$$

(51) $$\Psi_{2}=U_{S2_{1}}^{+}U_{S2_{2}}$$

$B_{1}(\theta ,\varphi ,\gamma ,\eta )$ and $B_{3}(\theta ,\varphi ,\gamma ,\eta )$ are of full rank, thus:

(52) $$\Phi_{i}=T_{i}\Psi_{i}T_{i}^{-1}\;(i=1,2)$$

This implies that the diagonal elements of the diagonal matrices $\Phi_{i}$ can be estimated by obtaining the K eigenvalues of each matrix $\Psi_{i}$, where the corresponding eigenvectors are the column vectors of $T_{i}$.

### 4.3. Polarization Estimation

It can be inferred from Equations (15) and (16) that:

(53) $$c_{xy}(\theta ,\varphi ,\gamma ,\eta )=c_{xx}(\theta ,\varphi ,\gamma ,\eta )P_{x}(\theta ,\varphi ,\gamma ,\eta )/P_{y}(\theta ,\varphi ,\gamma ,\eta )$$

The rotation invariant matrix can then be constructed by the above equation as:

(54) $$\Phi_{3}=\mathrm{diag}(P_{x}(\theta_{1},\varphi_{1},\gamma_{1},\eta_{1})/P_{y}(\theta_{1},\varphi_{1},\gamma_{1},\eta_{1}),\cdots ,P_{x}(\theta_{K},\varphi_{K},\gamma_{K},\eta_{K})/P_{y}(\theta_{K},\varphi_{K},\gamma_{K},\eta_{K}))$$

The matrix $C_{3}$ can be constructed using Equations (19) and (20) as:

(55) $$C_{3}=\left[\begin{array}{c} R_{4xx}\\ R_{4xy} \end{array}\right]=\left[\begin{array}{c} c_{xx}(\theta ,\varphi ,\gamma ,\eta )\\ c_{xx}(\theta ,\varphi ,\gamma ,\eta )\Phi_{3} \end{array}\right]C_{S}B^{H}(\theta ,\varphi ,\gamma ,\eta )$$

By using SVD, $C_{3}$ can be represented as:

(56) $$C_{3}=\left[\begin{array}{cc} U_{S3} & U_{N3} \end{array}\right]\left[\begin{array}{cc} \Sigma_{S3} & \\ & \Sigma_{N3} \end{array}\right]\left[\begin{array}{c} V_{S3}^{H}\\ V_{N3}^{H} \end{array}\right]$$

Based on the relationship between the subspace spanned by array steering vector and the signal subspace, we have:

(57) $$\left[\begin{array}{c} c_{xx}(\theta ,\varphi ,\gamma ,\eta )\\ c_{xy}(\theta ,\varphi ,\gamma ,\eta )\Phi_{3} \end{array}\right]T_{3}=\left[\begin{array}{c} U_{S3_{1}}\\ U_{S3_{2}} \end{array}\right]$$

The total least square solution of Equation (57) is:

(58) $$\Psi_{3}=U_{S3_{1}}^{+}U_{S3_{2}}$$

In the same way, $\Phi_{3}$ can be calculated by eigen-decomposition of $\Psi_{3}$.

### 4.4. Pair Matching

According to the discussion above, it is observed that the three eigen-decompositions of $\Psi_{i}$ are independent of each other. Therefore, pair matching is required when multiple signals impinge on the array. The matching process can be understood as the problem that whether any two parameters are successfully matched. Here we firstly consider the matching between $\Phi_{1}$ and $\Phi_{2}$. Parameters of one signal correspond to the same signal subspace, and the eigen-vector corresponding to different eigenvalues are mutually orthogonal. Therefore, a matrix can be constructed for ranking based on the following principle:

(59) $$H=T_{1}^{H}(i)T_{2}$$

where $T_{1}(i)$ denotes the i-th column of $T_{1}$. Therefore:

(60) $$j=\underset{j}{\min}\{Η(1),Η(2),\cdots ,Η(j),\cdots ,Η(K)\}$$

The pair matching between $\Phi_{1}$ and $\Phi_{3}$ is also similarly conducted. At the same time, K groups of values $u_{B1k}$, $u_{B3k}$ and ${\left(P_{x}/P_{y}\right)}_{k}$ are obtained , where k=1,2,⋯,K. The values of the azimuth angles, elevation angles, polarization auxiliary angles and polarization phase differences can be estimated as follows:

(61) $$\varphi_{k}=\arcsin\left(\frac{\lambda }{2\pi r}\sqrt{\left({\mathrm{angle}}^{2}(u_{B1})+{\mathrm{angle}}^{2}(u_{B3}))/(2-\sqrt{2}\right)}\right)$$

(62) $$\theta_{k}=\arctan\left(\frac{2-\sqrt{2}+\sqrt{2}\mathrm{angle}(u_{B1})/\mathrm{angle}(u_{B3})}{\sqrt{2}+(\sqrt{2}-1)\mathrm{angle}(u_{B1})/\mathrm{angle}(u_{B3})}\right)$$

(63) $$\gamma_{k}=\arctan\left(\left|\frac{\sin\theta_{k}+\mathrm{angle}({\left(P_{x}/P_{y}\right)}_{k})\cos\theta_{k}}{\cos\varphi_{k}\cos\theta_{k}-\mathrm{angle}({\left(P_{x}/P_{y}\right)}_{k})\cos\varphi_{k}\sin\theta_{k}}\right|\right)$$

(64) $$\eta_{k}=\mathrm{angle}\left(\frac{\sin\theta_{k}+\mathrm{angle}({\left(P_{x}/P_{y}\right)}_{k})\cos\theta_{k}}{\cos\varphi_{k}\cos\theta_{k}-\mathrm{angle}({\left(P_{x}/P_{y}\right)}_{k})\cos\varphi_{k}\sin\theta_{k}}\right)$$

### 4.5. Steps of the Proposed Algorithm

The steps of the proposed algorithm are executed as follows:

Step 1: Construct the fourth-order cumulant based on Equations (19) and (20).
Step 2: Select two pairs of sub-arrays which display rotational invariance based on the theory introduced in Section 4.1.
Step 3: Construct the rotational invariance matrices $C_{1}$, $C_{2}$ and $C_{3}$.
Step 4: Obtain $\Psi_{1}$, $\Psi_{2}$ and $\Psi_{3}$ based on the total least squares ESPRIT algorithm.
Step 5: Perform pair matching among $\Psi_{1}$, $\Psi_{2}$ and $\Psi_{3}$ and estimate the DOA and polarization information of the incident signals.

### 4.6. Computational Complexity

In the following discussion, we first derive the computational complexity of the proposed algorithm, and then compare the computational complexity with that of the LV-MUSIC algorithm. The main computational complexity of the proposed method is discussed as follows: (1) The calculation of the fourth-order cumulant slices requires $C_{1}=9L(M^{2}+2)$ complex multiplications. The calculation of one fourth-order cumulant requires 9L complex multiplications, and $M^{2}+2$ fourth-order cumulants are required for DOA and polarization estimation in the proposed algorithm; (2) SVD of matrices $C_{1}$, $C_{2}$ and $C_{3}$ requires $C_{2}=C_{3}=14(M^{2}+2)(K+2)$ and $C_{4}={\left(M^{2}+2\right)}^{2}(K+2)/2$ complex multiplications, respectively; (3) Solving $\Psi_{1}$, $\Psi_{2}$ and $\Psi_{3}$ using the total least squares requires $C_{5}=C_{6}=14K^{2}$ and $C_{7}=(M^{2}+2)K^{2}/2$ complex multiplications, respectively; (4) Eigen value decomposition (EVD) of matrices $\Psi_{1}$, $\Psi_{2}$ and $\Psi_{3}$ requires $C_{8}=3K^{2}(K+2)$ complex multiplications; (5) Pair matching of the parameters requires $C_{9}=2K^{3}$ complex multiplications.

The main contributors of computational complexity of LV-MUSIC method are discussed as follows: (1) The structure of the covariance matrix requires $C_{1}^{\prime }=LN^{2}=4LM^{2}$ complex multiplications; (2) The EVD of the covariance matrix requires $C_{2}^{\prime }=4M^{2}(K+2)$ complex multiplications; (3) Assuming that peak searching with all identical parameters, the construction of the spectrum function requires $C_{3}^{\prime }={\left(1+360/n\right)}^{2}{\left(1+90/n\right)}^{2}(4M^{2}+2M)$ complex multiplications, where each searching point of the spectrum function requires $C_{31}^{\prime }=N^{2}+N=4M^{2}+2M$ complex multiplications. 1+360/n is the number of searching points along the azimuth angle and polarization phase difference, 1+90/n is the number of searching points along the elevation angle and polarization auxiliary angle, and n is the searching step with respect to every parameter.

**Figure 6:**
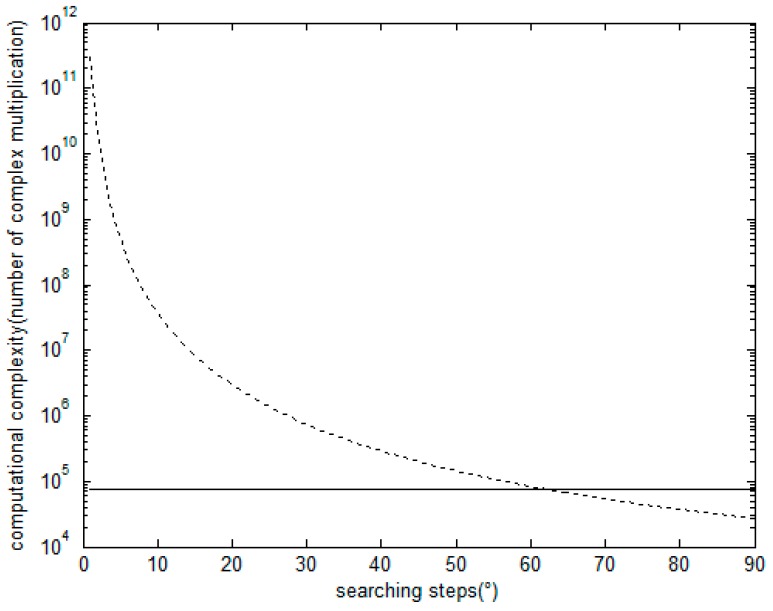
Computational complexity of the proposed algorithm and LV-MUSIC algorithm with respect to searching steps.

When the array structure, source number and snapshots are definite, it can be inferred from the analysis above that the computational complexity of the proposed algorithm is a fixed number, but that of the LV-MUSIC algorithm changes with searching steps. In this paper, we assume that the source number is 2 and the number of snapshots is 100. The computational complexity of the proposed algorithm and that of the LV-MUSIC algorithm using different searching steps are shown in Figure 6.

From Figure 6, it can be observed that the computational complexity of the LV-MUSIC algorithm is higher than that of the proposed algorithm when the searching step is less than 60°. The estimation accuracy of the LV-MUSIC algorithm is known to converge to half of the searching steps, and therefore the LV-MUSIC algorithm becomes invalid when the searching step is too large. In other words, though the LV-MUSIC algorithm can be used to estimate DOA and polarization jointly in theory, its computational complexity is not acceptable, mainly because the LV-MUSIC algorithm requires four dimensional peak searching. Fortunately, the proposed algorithm are not limited to these problems with a fixed computational complexity. The estimation accuracy and the performance of the proposed algorithm are discussed in the following section.

## 5. Simulation Results

In this section, several numerical simulation results are presented to illustrate the performance of the proposed algorithm. A uniform circular array with eight crossed dipoles elements and array radius of 0.087m is used in the simulations. Complex additive Gaussian white noise is added into the system and the number of Monte Carlo trials is 200.

The root mean square error (RMSE) for the estimated parameters is defined as:

(65) $$\mathrm{RMSE}=\sqrt{\frac{1}{M}\sum_{m=1}^{M}（{\hat{\chi }}_{im}-\chi_{i}{）}^{2}}$$

where M is the number of Monte Carlo trials, $\chi_{i}$ is the perfect value of the estimated parameter corresponding to the i-th incident signal, and ${\hat{\chi }}_{im}$ is the estimated value of the m-th time Monte Carlo trials.

### 5.1. The Simulation Results Distribution Scatter Diagram of the Proposed Algorithm

**Figure 7:**
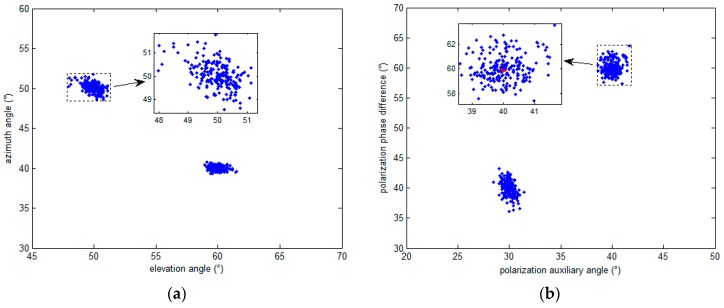
The simulation results distribution scatter diagram of proposed algorithm for (**a**) DOAs and (**b**) polarization parameters.

Figure 7 shows the simulation results for two signals whose DOAs and polarization parameters (θ,φ,γ,η) are $\left(60^{\circ },40^{\circ },30^{\circ },40^{\circ }\right)$ and $\left(50^{\circ },50^{\circ },40^{\circ },60^{\circ }\right)$, respectively. The proposed algorithm is carried out here, and the number of Monte Carlo trials is 200. Figure 7 shows the simulation result of all trials with the signal to noise ratio(SNR) of 20 dB, while the number of snapshots is 200. The red points in each figure represent the perfect value of the estimated parameter, while the blue points represent the estimated ones. The small windows inside the figures are the enlarged versions of the dotted box. Figure 7 shows that the points which represent the results of the proposed algorithm always cluster around the true value of the estimated parameters. From Figure 7a, it can be seen that the maximum evaluated error of elevation and azimuth angles are both less than 2°. From Figure 7b, we can see that the maximum evaluated error of polarization auxiliary angles and polarization phase differences are less than 2° and 3°, respectively. It can be observed that the proposed algorithm shows excellent performance.

### 5.2. Performance under Different SNR

**Figure 8:**
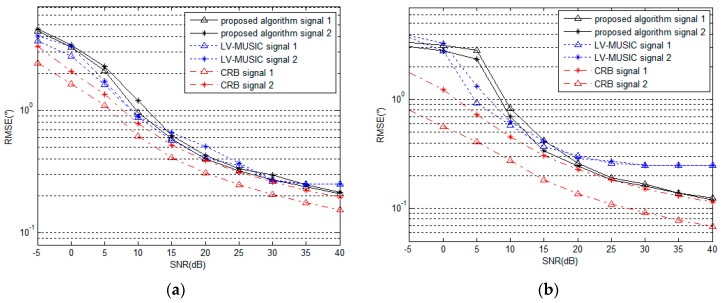
RMSE of (**a**) azimuth angle and (**b**) elevation angle estimation versus SNR (for 200 snapshots).

**Figure 9:**
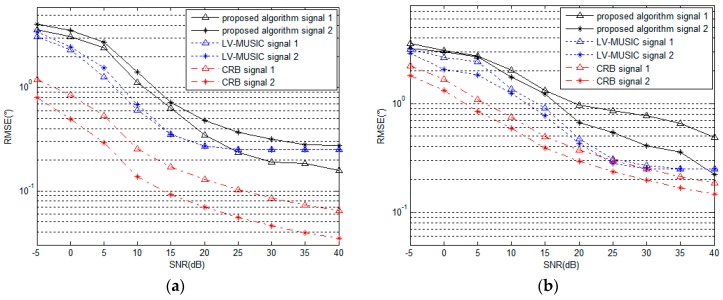
RMSE of (**a**) polarization auxiliary angle and (**b**) polarization phase difference estimation versus SNR (for 200 snapshots).

In order to demonstrate the remarkable performance of the proposed algorithm, we compare the performance of the proposed algorithm with that of the LV-MUSIC algorithm and the Cramer-Rao bound (CRB) with different values of SNR.As shown in Figure 6 above, the computational complexity of LV-MUSIC algorithm is huge when DOA and polarization parameters are estimated within acceptable searching steps. Due to limited computing power, when we focus on the DOA parameters estimation performance of LV-MUSIC algorithm, we suppose that the polarization parameters are known or they are estimated beforehand, and when we focus on the polarization parameters estimation performance of LV-MUSIC algorithm, the DOA parameters are set to be known or have been estimated already. Referring to this comparison, we assume that there are two signals with parameters (θ,φ,γ,η) of $\left(60^{\circ },40^{\circ },30^{\circ },40^{\circ }\right)$ and $\left(50^{\circ },50^{\circ },40^{\circ },60^{\circ }\right)$, the number of snapshots is 200, and the SNR changes from −5 dB to 40 dB. As shown in Figure 8 and Figure 9, it can be seen that as the SNR increases, the RMSE of the estimated parameters decreases gradually for the proposed algorithm, the LV-MUSIC algorithm and the corresponding CRB. Meanwhile, the proposed algorithm and the LV-MUSIC algorithm both achieve good DOA and polarization estimation performance, and the results also match with the CRB perfectly. The estimation accuracy of the proposed algorithm is similar to the LV-MUSIC algorithm. When the SNR increases, the RMSE of the proposed algorithm continuously decreases, and yet that of the LV-MUSIC algorithm remains unchanged, which depends on the searching step.

Comparison between Figure 8 and Figure 9 show that the RMSE of polarization parameters estimation is higher than that of DOA parameters estimation for the proposed algorithm for the same SNR. The reason is that the estimation of polarization parameters is carried out based on the estimated values of DOA parameters. The secondary error, thus, affects the estimation accuracy of polarization parameters.

### 5.3. Performance for Different Numbers of Snapshots

**Figure 10:**
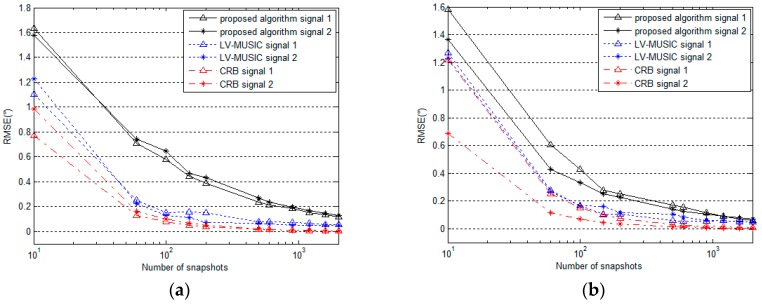
RMSE of (**a**) azimuth angle and (**b**) elevation angle estimation versus number of snapshots (for SNR = 20 dB).

**Figure 11:**
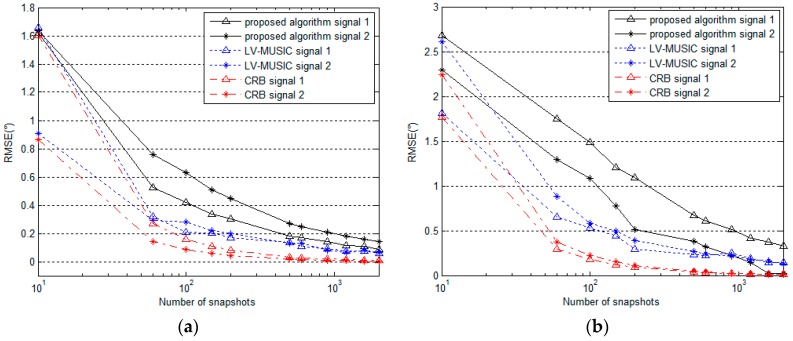
RMSE of (**a**) polarization auxiliary angle and (**b**) polarization phase difference estimation versus number of snapshots (for SNR = 20 dB).

The performance of the proposed algorithm is compared with the LV-MUSIC algorithm and the Cramer-Rao bound (CRB) versus different number of snapshots. Two signals with parameters (θ,φ,γ,η) of $\left(60^{\circ },40^{\circ },30^{\circ },40^{\circ }\right)$ and $\left(50^{\circ },50^{\circ },40^{\circ },60^{\circ }\right)$ are considered here, the SNR is set as 20 dB, and the number of snapshots changes from 10 to 2000. From Figure 10 and Figure 11, we can see that the RMSE values of these estimated parameters decrease gradually for the proposed algorithm, the LV-MUSIC algorithm and the CRB as the number of snapshots increases. The RMSE of the proposed algorithm is larger than that of the LV-MUSIC algorithm, and the two algorithms both show good DOA and polarization estimation performance and keep agreement with the CRB perfectly. Obviously, reducing computational complexity by using the proposed algorithm is at the expense of slightly worse performance as shown in the figure.

### 5.4. Running Time

The running time of the proposed algorithm is compared against that of the LV-MUSIC algorithm with two dimensional search. Two signals with parameters (θ,φ,γ,η) of $\left(60^{\circ },40^{\circ },30^{\circ },40^{\circ }\right)$ and $\left(50^{\circ },50^{\circ },40^{\circ },60^{\circ }\right)$ are considered here, the SNR is set as 20dBand the number of snapshots is 200. For the LV-MUSIC algorithm, the azimuth angle and the elevation angle have been searched in the range of $0^{\circ }$ to $360^{\circ }$ and $0^{\circ }$ to $90^{\circ }$ with an interval of $0.5^{\circ }$, respectively. 200 Monte Carlo simulations are carried out. Table 2 shows the average running time of these two algorithms. The running time of the proposed algorithm is shorter than that of the LV-MUSIC algorithm with two dimensional searching. In addition, four dimensional search is required when the LV-MUSIC algorithm is used to jointly estimate of DOA and polarization information. It is assumed that the polarization auxiliary angle and the polarization phase difference of the LV-MUSIC algorithm have been achieved by searching in the range of $0^{\circ }$ to $360^{\circ }$ and $0^{\circ }$ to $90^{\circ }$ with an interval of $0.5^{\circ }$, respectively. The running time of LV-MUSIC algorithm with four dimensional search is $(360^{\circ }/0.5^{\circ })(90^{\circ }/0.5^{\circ })=129,600$ times of the case of two dimensional search. Therefore, the running time of the LV-MUSIC algorithm with four dimensional search is unacceptable, and the proposed algorithm can obtain the four parameters quite efficiently.

## 6. Conclusions

A fourth-order cumulant-based ESPRIT algorithm is proposed in this paper, which can achieve the joint estimation of the DOA and polarization information based on a uniform circular EVSA. The proposed algorithm overcomes the limitation of the ESPRIT algorithm of failing in uniform circular EVSAs, and simultaneously achieves a significant reduction in terms of the computation cost. The fourth-order cumulant has been used to virtually extend the original array, and then construct a few sub-arrays with identical shapes. By matching two pairs of sub-arrays with the rotation-invariant structure, the estimation of DOA and polarization information can be carried out using the ESPRIT algorithm. As the algorithm does not require the construction of the spectrum function and does not resort to a multidimensional peak search, the estimation results can be achieved quite efficiently and also ensured the acceptable simulation accuracy. The reduction in computational complexity of the proposed algorithm has been illustrated by comparing against the LV-MUSIC algorithm for different searching steps theoretically and numerically. Numerical simulation results validate that the proposed algorithm has higher calculation efficiency than the LV-MUSIC algorithm. Future work may focus on utilizing a hypercomplex framework, such as a tensor, to re-establish the model of a four-order cumulant matrix, aiming at obtaining higher estimation accuracy in the direction finding algorithm.

## Figures and Tables

**Table 1:** Relationships between the elements of the virtual sub-arrays and the elements of matrix ***D***.

Sub-Array 1	Element of D	Sub-Array 2	Element of D	Sub-Array 3	Element of D	Sub-Array 4	Element of D
A1	D(5,1)	B1	D(4,1)	A1	D(5,1)	B3	D(6,3)
B2	D(5,2)	C2	D(4,2)	B2	D(5,2)	C3	D(5,3)
B5	D(8,5)	A1	D(5,1)	B4	D(7,4)	C4	D(6,4)
B8	D(8,3)	C1	D(3,1)	B7	D(7,2)	A1	D(5,1)
C3	D(5,3)	D3	D(4,3)	C2	D(4,2)	D3	D(4,3)
C5	D(7,5)	B4	D(7,4)	C5	D(7,5)	D5	D(6,5)
C6	D(8,6)	B6	D(6,1)	C7	D(7,1)	B6	D(6,1)
C8	D(8,2)	D1	D(2,1)	C8	D(8,2)	B8	D(8,3)
D4	D(5,4)	E4	D(4,4)	D1	D(2,1)	C1	D(3,1)
D5	D(6,5)	C4	D(6,4)	D2	D(4,2)	E3	D(3,3)
D7	D(8,7)	C7	D(7,1)	D6	D(7,6)	E6	D(6,6)
D8	D(8,1)	E1	D(1,1)	D7	D(8,7)	C6	D(8,6)
E5	D(5,5)	D4	D(5,4)	E2	D(2,2)	D2	D(4,2)
E8	D(8,8)	D8	D(8,1)	E7	D(7,7)	D6	D(7,6)

**Table 2:** The running time of two algorithms.

Algorithm	Time (s)
LV-MUSIC(two dimensional searching)	4.8942
Proposed Algorithm	3.7952
